# Persistent Morbillivirus Infection Leads to Altered Cortactin Distribution in Histiocytic Sarcoma Cells with Decreased Cellular Migration Capacity

**DOI:** 10.1371/journal.pone.0167517

**Published:** 2016-12-02

**Authors:** Vanessa Maria Pfankuche, Mohamed Sayed-Ahmed, Vanessa Bono Contioso, Ingo Spitzbarth, Karl Rohn, Reiner Ulrich, Ulrich Deschl, Arno Kalkuhl, Wolfgang Baumgärtner, Christina Puff

**Affiliations:** 1 Department of Pathology, University of Veterinary Medicine Hannover, Hannover, Germany; 2 Institute for Biometry, Epidemiology and Information Processing, University of Veterinary Medicine Hannover, Hannover, Germany; 3 Department of Non-Clinical Drug Safety, Boehringer Ingelheim Pharma GmbH&Co KG, Biberach (Riß), Germany; 4 Center for Systems Neuroscience, University of Veterinary Medicine Hannover, Hannover, Germany; George Mason University, UNITED STATES

## Abstract

Histiocytic sarcomas represent rare but fatal neoplasms in humans. Based on the absence of a commercially available human histiocytic sarcoma cell line the frequently affected dog displays a suitable translational model. Canine distemper virus, closely related to measles virus, is a highly promising candidate for oncolytic virotherapy. Therapeutic failures in patients are mostly associated with tumour invasion and metastasis often induced by misdirected cytoskeletal protein activities. Thus, the impact of persistent canine distemper virus infection on the cytoskeletal protein cortactin, which is frequently overexpressed in human cancers with poor prognosis, was investigated *in vitro* in a canine histiocytic sarcoma cell line (DH82). Though phagocytic activity, proliferation and apoptotic rate were unaltered, a significantly reduced migration activity compared to controls (6 hours and 1 day after seeding) accompanied by a decreased number of cortactin mRNA transcripts (1 day) was detected. Furthermore, persistently canine distemper virus infected DH82 cells showed a predominant diffuse intracytoplasmic cortactin distribution at 6 hours and 1 day compared to controls with a prominent membranous expression pattern (p ≤ 0.05). Summarized, persistent canine distemper virus infection induces reduced tumour cell migration associated with an altered intracellular cortactin distribution, indicating cytoskeletal changes as one of the major pathways of virus-associated inhibition of tumour spread.

## Introduction

Neoplastic disorders still represent one of the most common causes of death in humans as well as in companion animals such as dogs and cats [1,2]. Furthermore, despite a wide range of therapeutic approaches including surgery, chemo- and radiotherapy, many tumour types still possess a guarded to poor prognosis [3,4]. One example of such a neoplasm, with comparable short survival times in humans and dogs represents the histiocytic sarcoma [3,5,6]. This highly aggressive tumour type, occurring in a localised or disseminated variant, has a limited response to different conventional therapies including chemo- and radiotherapy, highlighting the need for new therapeutic approaches to overcome the current limitations of a palliative care in most cases [3,5,7,8]. Median survival of human patients suffering from histiocytic sarcoma with greatest tumour dimensions of more than 3.5 cm for example is not exceeding 6 months regardless of the therapy [4]. A promising new approach to overcome restricted therapeutic alternatives might be oncolytic virotherapy, based on the ability of several viruses to destroy cancer cells by simultaneous wide protection of non-transformed tissue [9]. For this purpose, members of many different virus families are currently investigated thoroughly in human medicine, resulting in several clinical trials [10]. Measles virus, a member of the family *Paramyxoviridae*, yielded promising results as a potential oncolytic virus by inducing the regression of human lymphoma-xenografts in immuno-deficient nude-mice [11]. A closely related, veterinary relevant virus is represented by canine distemper virus (CDV) [12]. It is an enveloped, negative orientated, single-stranded RNA virus, containing six structure proteins [12]. CDV is capable of infecting canine lymphoid cell lines, histiocytic sarcoma cell lines, such as DH82 cells, and neoplastic lymphocytes *in vitro*, commonly inducing apoptosis of tumour cells [13,14]. Recent studies suggest a decreased invasive and metastatic potential of persistently CDV-infected DH82 cells compared to non-infected controls [15]. This persistently with the vaccine strain Onderstepoort of CDV (CDV-Ond) infected DH82 cell line possesses a consistent high infection rate of more than 85% infected cells and thus, reveals a suitable model to study potentially CDV induced oncolytic mechanisms in histiocytic sarcomas [15]. Future therapeutic strategies might also include the use of acute CDV infections as well as transplantation of persistently CDV-infected DH82 cells as a permanent virus source *in vivo*. However, the field of oncolytic virotherapy is still in its infancy and many questions remain to be asked and answered. Especially the function and mode of action of different viruses still remain largely unknown [9]. The lack of a commercially available human histiocytic sarcoma cell line in contrast to its canine counter-part and the close relationship between measles and canine distemper virus highlight the present study design as a suitable translational model for further research and possible future therapeutic interventions of this devastating disease in humans [6,16].

A hallmark of many malignant neoplasms represents their ability to metastasize [17]. For this process as well as for many other developmental and functional mechanisms including invasion of adjacent tissues, cell motility represents one main basic requirement [18]. Cell motility is mostly based on changes in the cytoskeleton, which is crucially depending on members of the actin family [19,20]. The actin cytoskeleton is critical for various aspects of the cell motility process, including polarisation, leading edge protrusion and cellular contraction [21]. One member of the actin family, frequently overexpressed in multiple human tumours, represents cortactin [21,22]. Cortactin is an actin-binding protein and a substrate of the Src-kinase, being involved in mechanisms, such as cell migration, invasion, synaptogenesis, endocytosis, intercellular contacts and host-pathogen interactions [23]. The over-expression of cortactin in many different types of tumours is accompanied by an increased cell-migration activity and metastatic potential resulting in a worsened prognosis [22]. Cell migration often depends on the ability of cells to form actin-rich protrusions, called podosomes or invadopodia [23–27]. Invadopodia selectively appear in invasive cancer cells in comparison to non-invasive neoplasms and possess the ability to degrade the extracellular matrix. A decreased release of matrix metalloproteinases, which are important regulators of extracellular matrix metabolism, is reported for cells with a selective inhibition of cortactin [28]. In addition, a decreased number of invadopodia has been documented in head and neck squamous cell carcinoma cells treated with cortactin inhibiting RNA [29].

Another important and well-known mechanism that facilitates metastasis formation is represented by a phenomenon called epithelial-mesenchymal transition (EMT), and mainly applies to carcinomas [30]. However, in sarcomas an analogous phenomenon has been described and referred to mesenchymal-epithelial transition (MET), which is similarly involved in the formation of tumour metastases [31–33].

The aim of the present study was to determine the impact of CDV infection on cell migration of canine histiocytic sarcoma cells (DH82 cells) with special emphasis on cortactin expression, gene regulation and possible functional implications.

## Results

### Persistent CDV infection does not alter cellular proliferation, apoptosis and phagocytosis

**Fig 1:**
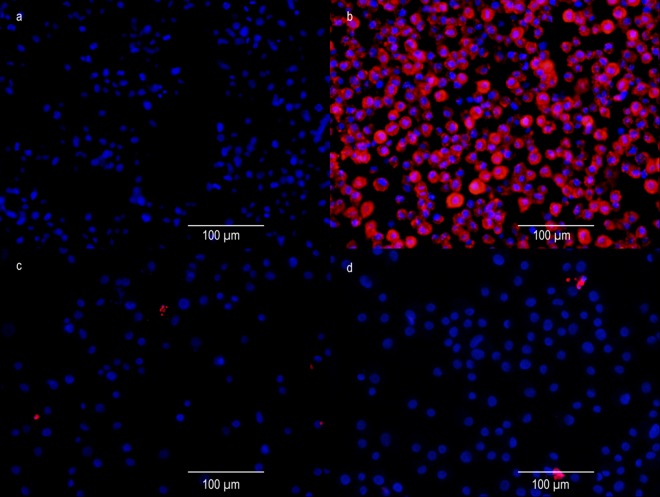
Immunofluorescence of non-infected DH82 cells and persistently CDV-Ond infected DH82 cells 1 day post seeding using anti-CDV nucleoprotein and anti-cleaved caspase 3 antibodies. (a) CDV-immunoreactivity is lacking in non-infected DH82 cells using an anti-CDV nucleoprotein antibody (D110; mouse monoclonal; 1:100; kind gift from Prof. Dr. A. Zurbriggen, University of Bern, Switzerland). (b) Immunofluorescence of persistently CDV-Ond infected DH82 cells using an anti-CDV nucleoprotein antibody (D110; mouse monoclonal; 1:100; kind gift from Prof. Dr. A. Zurbriggen, University of Bern, Switzerland) reveals a median percentage of 94.15% positive (bright red, secondary antibody: Cy™ 3-conjugated goat-anti-mouse IgG (H + L) antibody; 1:100; Jackson ImmunoResearch Laboratories, Hamburg, Germany) cells at 1d post seeding (minimum 92.99%; maximum 98.36%). Bisbenzimide was used for nuclear counterstaining (Sigma-Aldrich Chemie GmbH, Taufkirchen, Germany). (c, d) Immunofluorescence of non-infected and persistently CDV-Ond infected DH82 cells 1 day post seeding using an anti-cleaved caspase 3 antibody (Asp175; rabbit polyclonal; 1:900; Cat# 9661, RRID:AB_2341188; Cell Signaling Technology, Inc., Danvers, USA). Single immunopositive cells (bright red, secondary antibody: Cy™ 3-conjugated goat-anti-rabbit IgG (H + L) antibody; 1:100; Jackson ImmunoResearch Laboratories, Hamburg, Germany) are obvious in both non-infected (c) and CDV-infected cells (d) without significant differences between both conditions.

The persistent CDV infection of DH82 cells was ascertained using immunofluorescence for CDV nucleoprotein. Persistently CDV infected (strain Onderstepoort; CDV-Ond) DH82 cells exhibited a median percentage of infected cells of 94.15% at 1d post seeding (minimum 92.99%; maximum 98.36%; Fig 1A and 1B), 96.02% at 3d post seeding (minimum 93.95%; maximum 97.98%) and 94.58% at 5d post seeding (minimum 93.26%; maximum 98.16%), whereas no CDV-immunoreactivity was detected in non-infected controls at any time point (Fig 1A and 1B).

**Fig 2:**
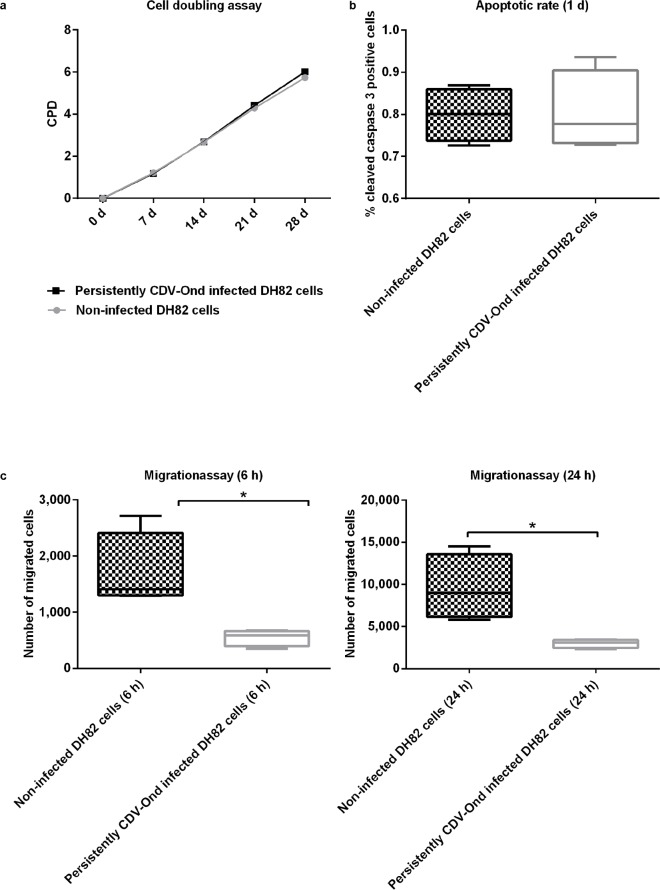
Overview of CDV induced changes on cell mechanisms including cell doubling, apoptosis and migration. (a) Persistent CDV-Ond infection of DH82 cells has no influence on cell proliferation as demonstrated by cells lacking a significant difference (p ≥ 0.05) in the cell doubling assay of non-infected and persistently infected DH82 cells. (b) Immunofluorescence of non-infected and persistently CDV infected DH82 cells reveals no significant difference (p ≥ 0.05) in the percentage of cleaved caspase 3 positive cells indicating a similar apoptotic rate following persistent CDV-Ond infection. Median, minimum and maximum percentages of immunopositive cells are presented. (c) Transwell migration assay of non-infected and persistently CDV-Ond infected DH82 cells reveals a significant difference (depicted by the asterisk, p ≤ 0.05) in the number of migrated cells 6h and 1d after seeding, thus indicating a reduced migratory activity of DH82 cells following persistent CDV-Ond infection. Median, minimum and maximum of counted cells are presented.

**Fig 3:**
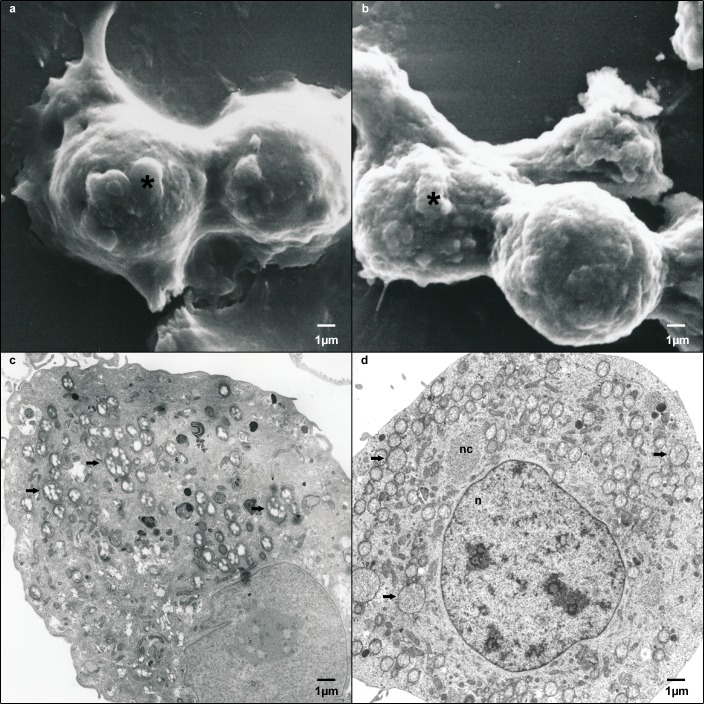
Scanning and transmission electron microscopy on non-infected and persistently CDV-Ond infected DH82 cells. Scanning and transmission electron microscopy was performed on non-infected and persistently CDV-Ond infected DH82 cells demonstrating comparable latex bead phagocytosis in both cell lines. Transmission electron microscopy reveals intracytoplasmic latex beads (arrows), the CDV nucleocapsid in persistently CDV-Ond infected DH82 cells (nc), the nuclei (n) and cellular protrusions (*). (a) Scanning electron microscopy on non-infected DH82 cells. (b) Scanning electron microscopy on persistently CDV-Ond infected DH82 cells. (c) Transmission electron microscopy on non-infected DH82 cells. (d) Transmission electron microscopy on persistently CDV-Ond infected DH82 cells.

Cumulative population doubling assay, performed for 4 weeks, revealed no significant differences in the proliferation rates of non-infected and persistently CDV-Ond-infected DH82 cells (p ≥ 0.05; Fig 2A). Similarly, the apoptotic rate as determined by cleaved caspase 3 immunofluorescence, showed no significant difference at 1 day post seeding (1d; p ≥ 0.05; Figs 1C and 1D; 2B). A median of 0.80% (minimum 0.73%; maximum 0.87%) of non-infected and 0.78% (minimum 0.73%; maximum 0.94%) of persistently CDV-Ond-infected DH82 cells was observed. Additionally, the detection of DNA strand breaks, indicating apoptotic cells, using terminal deoxynucleotidyl transferase-mediated dUTP-biotin nick end labeling (TUNEL), revealed no significant difference at 1 day post seeding (p ≥ 0.05) with a median of 1.20% (minimum 0.00%; maximum 3.20%) of non-infected and 2.00% (minimum 0.00%; maximum 2.4%) of persistently CDV-Ond-infected DH82 cells. Furthermore, typical macrophage functions, examined by scanning and transmission electron microscopy performing latex bead phagocytosis assay, were retained by persistently CDV-Ond infected DH82 cells independent of time point post seeding (Fig 3).

### Persistent CDV infection reduces the migration capacity of DH82 cells

At 6 hours post seeding (6h) in median 1411 non-infected DH82 cells reached the lower compartment of the transwell system (minimum 1290 cells; maximum 2719 cells). In contrast, persistently CDV-Ond infected DH82 cells displayed a median of 587 migrated cells at 6h (minimum 348 cells; maximum 677; p ≤ 0.05; Fig 2C), indicating a major impact of virus infection on cell migration. This was substantiated by the observation that at 1d after seeding in median 8995.5 migrated non-infected DH82 cells were found in the lower compartment (minimum 5806 cells; maximum 14502 cells), whereas only a median of 3068.5 persistently CDV-Ond infected DH82 cells (minimum 2328 cells; maximum 3441 cells) were migrated (p ≤ 0.05; Fig 2C).

### Microarray analysis reveals significant differences in the expression of invadopodia associated genes in DH82 cells by persistent CDV infection

A microarray dataset of non-infected and persistently CDV-Ond infected DH82 cells was analysed for changes by investigating the expression of a manually generated list of 77 literature based genes, which are known to be involved in invadopodia formation and function and thus influencing the actin cytoskeleton. Persistent CDV infection caused a significant difference (Mann Whitney U Test p ≤ 0.05 and fold change ≤ -2 or ≥ 2) in the expression of a total number of 12 unique canine gene symbols (5 down- and 7 up-regulated) out of 77 canine gene symbols (Table 1). Differentially expressed down-regulated genes comprised CTTN (cortactin), CAV1 (caveolin 1), PTK2 (PTK2 protein tyrosine kinase 2), TGFB2 (transforming growth factor, beta 2) and IQGAP2 (IQ motif containing GTPase activating protein 2) with fold changes ranging from -6.52 to -3.13 with cortactin as the top hit of down-regulated genes associated with invadopodia. Differentially expressed up-regulated genes comprised PDGFC (platelet derived growth factor C), MMP14 (matrix metallopeptidase 14), PIK3CG (phosphoinositide-3-kinase, catalytic, gamma polypeptide), FSCN1 (fascin homolog 1, actin-bundling protein), PIK3CD (phosphoinositide-3-kinase, catalytic, delta polypeptide), PDGFA (platelet-derived growth factor alpha polypeptide) and PLAUR (plasminogen activator, urokinase receptor) with fold changes ranging from 6.58 to 2.02.

**Table 1:** Persistent CDV infection causes a significant difference (Wilcoxon-Mann-Whitney test p ≤ 0.05 and fold change ≤ -2 or ≥ 2) in the expression of genes associated with invadopodia formation.

Canine gene symbol	Gene Title (*Canis familiaris*)	p-value	Fold change
CTTN	cortactin	0.029	-6.522
CAV1	caveolin 1, caveolae protein, 22kDa	0.029	-3.382
PTK2	PTK2 protein tyrosine kinase 2	0.029	-3.226
TGFB2	transforming growth factor, beta 2	0.029	-3.204
IQGAP2	IQ motif containing GTPase activating protein 2	0.029	-3.134
PLAUR	plasminogen activator, urokinase receptor	0.029	2.024
PDGFA	platelet-derived growth factor alpha polypeptide	0.029	2.089
PIK3CD	phosphoinositide-3-kinase, catalytic, delta polypeptide	0.029	2.134
FSCN1	fascin homolog 1, actin-bundling protein (Strongylocentrotus purpuratus)	0.029	2.483
PIK3CG	phosphoinositide-3-kinase, catalytic, gamma polypeptide	0.029	3.168
MMP14	matrix metallopeptidase 14 (membrane-inserted)	0.029	4.558
PDGFC	platelet derived growth factor C	0.029	6.578

Persistent CDV infection causes a significant difference (Wilcoxon-Mann-Whitney test p ≤ 0.05 and fold change ≤ -2 or ≥ 2) in the expression of a total number of 12 unique canine gene symbols (5 down- and 7 up-regulated) out of 77 manually selected literature based genes (S1 Table), which are known to be involved in invadopodia formation and function. Microarray analysis of these genes, revealed a significant, more than six-fold down-regulation of cortactin in persistently CDV-Ond infected DH82 cells compared to controls.

**Fig 4:**
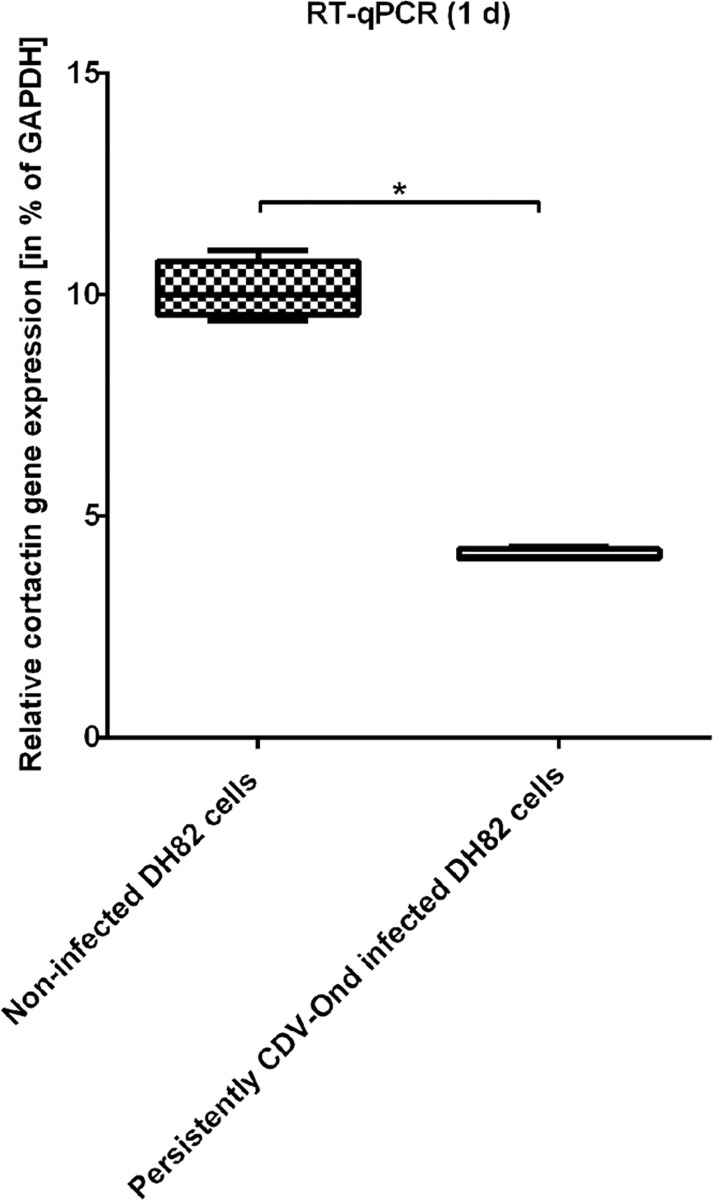
RT-qPCR of non-infected and persistently CDV-Ond infected DH82 cells. RT-qPCR confirms the significant difference in the relative cortactin gene expression in non-infected and persistently CDV-Ond infected DH82 cells as demonstrated by microarray analysis. The relative cortactin gene expression is calculated by normalisation against the housekeeping gene GAPDH. The relative percentage of target-specific gene expression was calculated as follows: X/Y × 100 = normalised target specific gene expression, where X = target-specific gene expression level and Y = housekeeping gene (GAPDH) expression level. (significance (p ≤ 0.05) is highlighted by an asterisk).

To substantiate these findings, the number of cortactin mRNA transcripts was determined using real-time quantitative PCR. At 1d post seeding, persistently CDV-Ond infected DH82 cells possessed significant lower numbers of cortactin mRNA transcripts compared to non-infected controls (p ≤ 0.001; Fig 4).

### Cortactin displays a predominantly, diffuse distribution in persistently CDV-Ond infected DH82 cells at early time points

**Fig 5:**
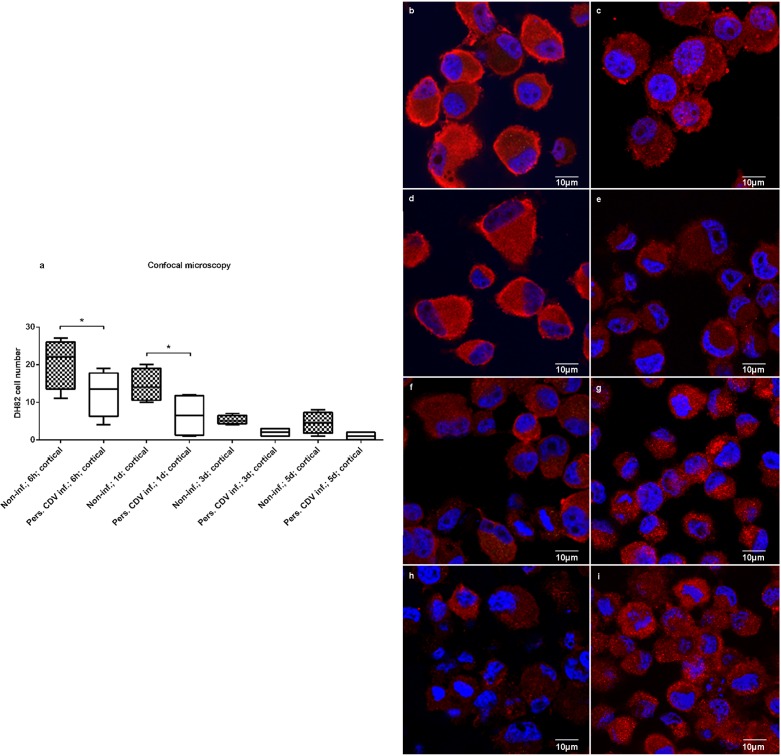
Confocal laser microscopy of non-infected and persistently CDV-Ond infected DH82 cells. (a) Confocal laser microscopy of non-infected and persistently CDV-Ond infected DH82 cells demonstrates significant differences in the intracellular cortactin distribution (significance is highlighted by asterisks). Depicted are differences in cortical cortactin distribution between non-infected and persistently CDV-Ond infected DH82 cells at the same time point (Figs 5 b-i). Immunofluorescence of non-infected (Figs 5b,d,f,h) and persistently CDV-Ond infected (Figs 5c,e,g,i) DH82 cells, 6h (Figs 5b,c), 1d (Figs 5d,e), 3d (Figs 5f,g) and 5d (Figs 5h,i) after seeding (red: cortactin; blue: nuclear staining with bisbenzimide) highlights the differences in the cortactin distribution pattern and the tendency of both cell types to exhibit a diffuse cytoplasmic cortactin distribution at later time points.

More than 90% of non-infected and persistently CDV-Ond infected DH82 cells were immunopositive for cortactin at all time points investigated (1d, 3d, 5d after seeding; p ≥ 0.01; Table 2). Interestingly, the intracellular cortactin distribution differed in persistently CDV-Ond infected DH82 cells at different time points compared to non-infected cells (Table 3; Fig 5). At 6h and 1d post seeding a significantly higher number of non-infected DH82 cells displayed a cortical cortactin expression compared to persistently CDV-infected DH82 cells (p ≤ 0.01). In addition, the percentage of cells with a cortical cortactin expression was higher in non-infected controls at all time points investigated, compared to persistently CDV-Ond infected DH82 cells, albeit not reaching the level of significance at 3 and 5 days post seeding (3d; 5d; p ≥ 0.05). Furthermore the percentage of cells with a cortical cortactin expression decreased over time in culture, independently of the virus-infection.

**Table 2:** Percentage of cortactin expression in non-infected (DH82) and persistently CDV-Ond infected (DH82pi) DH82 cells at different time points post seeding.

Cell type	Time point post seeding		
	1d	3d	5d
**DH82**	95.13%	96.26%	98.54%
	(94.66%; 96.82%)	(95.88%; 97.72%)	(98.27%; 98.57%)
**DH82pi**	98.09%	96.46%	98.39%
	(95.15%; 99.17%)	(95.21%; 97.13%)	(97.71%; 98.83%)
**p-value**	≥ 0.01	≥ 0.01	≥ 0.01

The values are shown as median (minimum; maximum). Statistically significant differences (p ≤ 0.01) between DH82 and DH82pi cells were not observed.

**Table 3:** Percentage of cortical cortactin expression in non-infected (DH82) and persistently CDV-Ond infected (DH82pi) cells at different time points post seeding.

Cell type	Time point post seeding			
	6h	1d	3d	5d
**DH82**	73.34%	46.67%	16.67%	15.00%
	(36.67%; 90.00%)	(33.33%; 66.67%)	(13.33%; 23.33%)	(3.33%; 26.67%)
**DH82pi**	45.00%	21.67%	6.67%	3.34%
	(13.33%; 63.33%)	(3.33%; 40.00%)	(3.33%; 10.00%)	(0.00%; 6.67%)
**p-value**	0.0042	0.0042	0.2282	0.1924

The values are shown as median (minimum; maximum). Statistically significant differences in the percentage of cells with a cortical cortactin expression between DH82 and DH82pi cells are highlighted in grey (p ≤ 0.05).

### Microarray analysis revealed significant differences in the expression of several genes involved in epithelial-mesenchymal transition (EMT) and mesenchymal-epithelial transition (MET) in DH82 cells by persistent CDV infection

A list of 32 literature based genes, which are known to be involved in EMT and MET, respectively, was manually generated in order to investigate potential changes in their expression in a microarray dataset of non-infected and persistently CDV-Ond infected DH82 cells. 6 out of 32 unique canine gene symbols (Table 4; S2 Table), from which 5 were up- and 1 down-regulated, showed a significant differential regulation in persistently infected cells compared to non-infected cells (Mann Whitney U Test p ≤ 0.05 and fold change ≤ -2 or ≥ 2). In particular, LEF1 (lymphoid enhancer-binding factor 1) with a fold change of -2.292 represented the only differentially expressed down-regulated gene, whereas TRPS1 (trichorhinophalangeal syndrome I), CD44 [CD44 molecule (Indian blood group)], HGF [hepatocyte growth factor (hepapoietin A; scatter factor)], TWIST1 [twist homolog 1 (Drosophila)], CDH2 [cadherin 2, type 1, N-cadherin (neuronal)] were up-regulated with fold changes ranging from 2.092 to 77.297.

**Table 4:** Persistent CDV infection of DH82 cells causes a significant differential regulation of genes associated with epithelial-mesenchymal transition (EMT) and mesenchymal-epithelial transition (MET), as determined by Wilcoxon-Mann-Whitney test p ≤ 0.05 and fold change cut-off ≤ -2 or ≥ 2.

Canine gene symbol	Gene Title (*Canis familiaris*)	p-value	Fold change
LEF1	lymphoid enhancer-binding factor 1	0.029	-2.292
TRPS1	trichorhinophalangeal syndrome I	0.029	2.092
CD44	CD44 molecule (Indian blood group)	0.029	2.657
HGF	hepatocyte growth factor (hepapoietin A; scatter factor)	0.029	2.766
TWIST1	twist homolog 1 (Drosophila)	0.029	3.035
CDH2	cadherin 2, type 1, N-cadherin (neuronal)	0.029	77.297

## Discussion

The hallmark of many malignant tumours is their ability to invade the adjacent tissue and to form metastases [34]. Often these factors dramatically influence the prognosis for the affected individual. For invasion and metastasis neoplastic cells need the ability to migrate through the adjacent extracellular matrix, which is commonly mediated by the formation of invadopodia [35]. Therefore inhibition of invadopodia formation with consecutive reduction of tumour cell migration represents an exciting new possibility for improving prognosis and survival time. One possibility of targeting invadopodia include the administration of different drugs often targeting pathways like the Src signalling, platelet derived growth factor signalling pathway and metalloprotease activity [36]. However, effective therapeutic drugs targeting these pathways, for example the Src signalling, are often lacking or only useful at an early developmental stage [36], demonstrating the need of new treatment strategies like viral oncolysis.

Viral oncolysis, especially with morbilliviruses like measles virus and canine distemper virus, might represent an elegant method to overcome the common limitations of chemotherapy since many studies have shown that these viruses predominantly infect neoplastic cells while largely sparing non-transformed tissue [10]. Furthermore, despite the existence of several genetically modified virus strains, many life-attenuated vaccine strains, often known and well tolerated since decades, have been shown to exert oncolytic activity *in vitro* and *in vivo* [10,37].

While depicting very similar proliferation and apoptotic rates as well as retaining phagocytic activity as a typical macrophage function, DH82 cells persistently infected with CDV (vaccine strain Onderstepoort) and non-infected controls differed significantly in their migratory capacity with significantly lower numbers of migrated virus-infected DH82 cells compared to non-infected controls in the present study. Interestingly, measles virus has been shown to interact with the Src-kinase pathway, which is also involved in invadopodia formation [38]. In addition, canine distemper virus is known to interfere with the actin cytoskeleton [39–41]. Therefore the present study focused on the influence of CDV-infection upon the cytoskeleton, namely constituents of invadopodia formation.

Microarray analysis of genes, associated with invadopodia formation and thus also partially involved in the actin cytoskeleton, revealed a modulation of several genes. Besides cortactin, several actin-related molecules have been shown to be involved in invadopodia formation [25,42–46]. However, with the striking exception of cortactin the majority of actin-related genes included in the gene list used in the present study (S1 Table) did not reveal differential regulation. Interestingly, a significant, more than six-fold down-regulation of cortactin in persistently infected DH82 cells compared to controls was observed, which was further substantiated by using quantitative PCR. Cortactin represents an actin regulator required for invadopodia formation and also a substrate of the Src-kinase [42,47]. Furthermore, several studies demonstrated a correlation between a high cortactin expression and a poor prognosis in several types of human neoplasms such as oesophageal squamous cell carcinoma, pancreatic and colorectal adenocarcinoma and laryngeal carcinoma [48–51]. This implicates that a strategy to reduce the cortactin expression in other malignant neoplasms might also be helpful to improve prognosis and survival time. However, the influence of modulating the cortactin expression in histiocytic sarcomas has not been investigated so far, despite the fact that histiocytic sarcoma cells link both, a cell type which typically is able to migrate and a malignant transformation of the cells. Mesenchymal-epithelial transition (MET) of mesenchymal tumour cells represents another mechanism that facilitates metastasis formation. Microarray analysis of MET-associated genes revealed a modulation of 6 out of 32 genes, thus representing a potential additional effect of CDV-Ond infection, which might play a role as a contributing factor that explains the observed phenotypical decrease in the migration activity of CDV-Ond infected DH82 cells. Earlier studies have shown that expression of genes encoding intercellular and cell-to-extracellular matrix adhesion molecules is altered in some highly aggressive carcinomas as well as sarcomas [32]. Indeed, several adhesion molecules were altered in the present study (S2 Table) with CDH2 [cadherin 2, type 1, N-cadherin (neuronal)] as the gene with highest differential expression (Wilcoxon-Mann-Whitney test p ≤ 0.05 and fold change ≤ -2 or ≥ 2). CDH2 is markedly up-regulated in persistently infected DH82 cells, suggesting an influence of a CDV infection on molecules involved in intercellular adhesion and thus also in EMT and MET by different pathways including the well-described phenomenon of a cadherin switch [52]. The cadherin switch describes the loss of E-cadherin followed by an overexpression of n-cadherin which results in tumour progression and metastasis of several epithelial tumors [53]. Nevertheless, in the present study, an up-regulation of CDH2 is accompanied by a migration-inhibited phenotype of DH82 cells.

E-cadherin has previously been demonstrated to play a fundamental role as a suppressor of migration, invasion and metastasis in carcinomas [54]; however, even though this molecule was included in the list of genes (S2 Table) it did not reach the level of significance based on the used filtering criteria.

Nevertheless, further studies should be undertaken to elucidate the role of n- and e-cadherin in oncolytic mechanisms of CDV in histiocytic sarcoma.

The present study was conducted using histiocytic sarcoma cells, a tumour type with comparable poor prognosis in humans and dogs [3–5,55]. The canine tumour cell line (DH82 cells) was chosen as a translational model with benefits for both, humans and dogs, since no permanent human histiocytic sarcoma cell line is commercially available [16,56]. Moreover, presented oncolytic effects of a CDV infection in DH82 cells were demonstrated in a cell line with a constant high infection rate. Data subsequently could be used for further *in vivo* and *in vitro* studies in the canine translational model and their extrapolation to the human counter-part with respect to tumour type and morbillivirus to be applied.

In contrast to the significant down-regulation of cortactin in persistently CDV-Ond infected DH82 cells on a molecular level, the number of cortactin expressing cells did not differ significantly between persistently CDV-Ond infected DH82 cells compared to controls on the protein level at any time point investigated. Similarly it has been described for different breast cancer cell lines, that the expression level of cortactin does not directly correlate with the ability of cells to form invadopodia and to migrate [57], stressing the importance of the subcellular localisation of cortactin.

The present study revealed significant differences in the intracellular localisation of cortactin between non-infected and persistently CDV-Ond infected DH82 cells at early time points (6h and 1d post seeding). At these time points cortactin was mainly located beneath the cell membrane and within cellular processes in non-infected cells, whereas persistently CDV-Ond infected DH82 cells exhibited a diffuse cytoplasmic distribution. As a membrane-associated cortactin expression is attributed to an “active” state with invadopodia formation and migration [58] the diffuse distribution in persistently CDV-Ond infected DH82 cells might explain their reduced locomotion. A similar observation, also emphasising the importance of the intracellular cortactin localisation, has been made in human fibrosarcoma cells, where the total amount of cortactin was unchanged by treatment with alpha-tocopheryl phosphate, whereas a delocalisation of cortactin from cell membrane and invadopodia to the cytoplasm occurred accompanied by reduced cell motility [58]. The enhanced diffuse cytoplasmic distribution of cortactin at later time points (3d and 5d post seeding) in both CDV-Ond infected and non-infected DH82 cells was attributed to the increased confluence of cultures, since a contact inhibition of cell migration associated with cell density has been described for many cell types including cancer cells [59–61].

Summarised, persistent CDV-Ond infection of canine histiocytic sarcoma cells reduced the cellular migration capacity *in vitro*, associated with a diminished cortactin accumulation at the cell periphery. This might indicate a reduced metastatic potential of CDV infected DH82 cells *in vivo*. However, the latter has to be substantiated in further *in vivo* studies. Additionally, this canine model of viral oncolysis might represent an interesting translational method for this rare human tumour since CDV represents the canine counterpart of the closely related measles virus.

## Materials and Methods

### Cell culture

DH82 cells are a permanent canine histiocytic sarcoma cell line, obtained from the European Collection of Cell Cultures (ECACC No. 94062922), originally isolated from a Golden Retriever [56]. DH82 cells were cultivated as formerly described [15]. Passage 10 of non-infected and passage 141 of persistently CDV-infected DH82 cells were used for the present experiments. Persistently CDV-infected DH82 cells were generated as described [15]. Cells were periodically harvested, frozen and stored in liquid nitrogen.

### Cell doubling assay

To assess cellular proliferation, the cumulative population doubling (CPDs) was determined. During continuous passages, cells were seeded at same numbers into 25 cm^2^ tissue culture flasks (Nunc GmbH & Co. KG, Thermo Scientific, Langenselbold, Germany) and counted at each, weekly passage over 4 weeks. The population doubling (PD) was calculated according to the following formula:

“PD = log_10_ (cells harvested—initial cell number) / log_2_”. The cumulative population doubling was performed by adding the population doubling of each passage to that of the previous passage [62,63].

### Migration assay and cytospin preparation

The migration ability of non-infected and persistently CDV-Ond infected DH82 cells was assessed by transwell migration assays. Cells were seeded on uncoated 24-well Millicell cell culture inserts with a pore diameter of 8μm (Merck KGaA, Darmstadt, Germany) at a density of 200000 cells/well in minimal essential medium (MEM) with Earles´s salts (PAA, Cölbe, Germany), 1% penicillin/streptomycin (P/S; PAA) and 1% non-essential amino acids (NEAA; Sigma-Aldrich Chemie GmbH, Taufkirchen, Germany). The lower chamber additionally contained 10% foetal calf serum (FCS; PAA) as a chemoattractant. The transwell systems were incubated at 37°C, 5% CO_2_ in a water-saturated atmosphere for 6 and 24 hours, respectively. Migrated cells were scraped, re-suspended and cytospin preparations were performed. After Pappenheim staining all migrated cells were counted.

### Immunofluorescence

Non-infected and persistently CDV-Ond infected DH82 cells were stained for cleaved caspase 3 (Asp175; rabbit polyclonal; 1:900; Cat# 9661, RRID:AB_2341188; Cell Signaling Technology, Inc., Danvers, USA) with a secondary Cy3-conjugated goat-anti-rabbit IgG (H+L) antibody (1:100; Cat# 111-165-144, RRID:AB_2338006; Jackson ImmunoResearch Laboratories, Hamburg, Germany) to determine the number of apoptotic cells 1d after seeding. Nuclear staining was performed with bisbenzimide (Hoechst 33258; Sigma-Aldrich Chemie GmbH). Briefly, cells were seeded in quadruplicates at a density of 30000 cells/cm^2^ on 96 Microwell Nunc plates (Nunc GmbH & Co. KG, Thermo Scientific) and maintained under standard conditions. Cells were fixed with 4% paraformaldehyde at 1d after seeding and immunofluorescence was performed according to a 2 day protocol with minor variations [64].

The number of cortactin positive cells (1d, 3d and 5d) and the intracellular cortactin distribution (6h, 1d, 3d and 5d) were determined using a polyclonal anti-cortactin IgG antibody (H-191; rabbit polyclonal; diluted 1:100; Cat# sc-11408, RRID:AB_2088281; Santa Cruz Biotechnology, California, USA). To verify the CDV infection of persistently CDV-Ond infected DH82 cells on a cellular level double-labelling with an anti-CDV nucleoprotein antibody (D110; mouse monoclonal; 1:100; kind gift from Prof. Dr. A. Zurbriggen, University of Bern, Switzerland) was performed. As secondary antibodies a Cy3-conjugated goat-anti-rabbit IgG (1:100; Cat# 111-165-144, RRID:AB_2338006; Jackson ImmunoResearch Laboratories, Hamburg, Germany) and an Alexa Fluor 488-conjugated AffiniPure goat-anti-mouse IgG (H+L) antibody (dilution 1:200; Cat# 115-545-003, RRID:AB_2338840; Jackson ImmunoResearch Laboratories) were used. Cells were seeded in quadruplicates at a density of 30000 cells/well on 96 Microwell Nunc plates (number of cortactin positive cells) and at a density of 50000 cells/well in 8 well lab-Tek chamber slides (intracellular cortactin distribution; Nunc GmbH & Co. KG, Thermo Scientific).

### Terminal deoxynucleotidyl transferase-mediated dUTP-biotin nick end labeling (TUNEL) for the detection of DNA strand breaks

Non-infected and persistently CDV-Ond infected DH82 cells were seeded in quadruplicates at a density of 100000 cells/well in 4 well lab-Tek chamber slides (Nunc GmbH & Co. KG, Thermo Scientific). One day post seeding, detection of DNA strand breaks was performed by terminal deoxynucleotidyl transferase-mediated dUTP-biotin nick end labeling (TUNEL) using the ApopTaq Plus peroxidase *in situ* apoptosis detection kit (S7101, EMD Millipore Corporation, Temecula, USA) according to the manufacturer's instructions as formerly described [65].

### Laser scanning confocal microscopy

The intracellular cortactin distribution was analysed in detail by laser scanning confocal microscopy using the Leica TCS SP5 AOBS with a tandem-scanner and the Leica Application Suite Advanced Fluorescent Lite 2.0.2 build 2038 (Leica, Biberach, Germany). To evaluate the intracellular cortactin distribution three-dimensionally, 0.5μm thick z-stacks were recorded. The staining pattern was categorized as cell-membrane / protrusion accentuated (cortical) or diffuse cytoplasmic. Cells displaying a cortical cortactin staining in at least one half (≥ 50%) of the cellular circumference in at least one z-stack were defined as cells with a cortical staining pattern, whereas all others were described as diffusely cytoplasmic. 30 cells of each well (n = 4) and each condition were analysed per time point and classified.

### Phagocytosis assay

Phagocytic activity of non-infected and persistently CDV-Ond infected DH82 cells was determined using polystyrene latex beads (0.8μm; Sigma-Aldrich Chemie GmbH, Taufkirchen, Germany) in a dilution of 1:100 in MEM (PAA) containing 1% P/S (100 units/ml, 100 mg/ml; PAA), 10% FCS (PAA) and 1% NEAA (Sigma-Aldrich Chemie GmbH). 250μl of this solution were added to each well in a 24 well plate (Nunc GmbH & Co. KG, Thermo Scientific) or 4 well lab-Tek chamber slides (Nunc GmbH & Co. KG, Thermo Scientific) containing non-infected and persistently CDV-Ond infected DH82 cells 1d, 3d and 5d post seeding. Cells were incubated for 3 hours at 37°C and 5% CO_2_ in a water-saturated atmosphere. Afterwards, cells were washed twice with phosphate buffered saline (PBS) to remove excess latex beads. The presence of phagocytized particles was determined by scanning and transmission electron microscopy [66].

### Transmission electron microscopy

Embedding and sample processing were performed as described before [67,68]. Transmission electron microscopic analysis was performed by a transmission electron microscope (EM C 10A, Zeiss, Jena, Germany) at 60kV.

### Scanning electron microscopy

Embedding and sample processing was performed as described previously [69]. Slides were examined with a scanning electron microscope (DSM940, Zeiss).

### RNA isolation and cDNA synthesis

RNA isolation was performed as described previously [15]. RNA concentration was ascertained by measuring the optical density at 260nm. The Omniscript kit (Qiagen N. V., Venlo, The Netherlands) with RNase Out (Invitrogen™ GmbH, Darmstadt, Germany) and random hexamers (Random Primers, Promega, Fitchburg, USA) was used for reverse transcription of total RNA into complementary DNA (cDNA) following the manufacturers’ protocol.

### Primer design

Primers used in this study were designed with Primer3 software [70], Beacon Designer version 2.1 software (Premier Biosoft International, Palo Alto, USA) or taken from the literature [15]. Primer oligonucleotides were purchased from Eurofins MWG Operon (Ebersberg, Germany).

### RT-PCR

RT-PCR was performed using a PTC200 thermocycler (Biozym, Hessisch Oldendorf, Germany) under the following conditions: 94°C for 1min, 40 cycles at 94°C for 1min, 58°C (GAPDH) or 59°C (cortactin) for 2min, 72°C for 1min and 72°C for 5min. Amplification was performed using AmpliTaq DNA Polymerase (Applied Biosystems Applera Deutschland GmbH, Darmstadt, Germany) in 1x GeneAmp 10x PCR Buffer II (Applied Biosystems Applera Deutschland GmbH) with 1.25 mM MgCl_2_, 0.2 mM dNTP mix (Applied Biosystems Applera Deutschland GmbH) and 300 nM of each primer [Cortactin: Forward: 5’-GACTGGGAGACTGACCCTGA-3’; Reverse: 5’-ACACCAAACTTGCCTCCAAA-3’; 320 base pairs (bp); GenBank accession number: XM_005631371; glyceraldehyde-3-phosphate-dehydrogenase (GAPDH): Forward: 5‘-AAGGTCGGAGTCAACGGATT-3‘; Reverse: 5‘-GCAGAAGAAGCAGAGATGATG-3‘; 365 bp; GenBank accession number: AB038240]. PCR products were analysed by agarose gel electrophoresis.

### Real-time quantitative PCR

Real time quantitative PCR (RT-qPCR) was performed as described [15]. In addition to cDNA samples, tenfold serial dilutions of purified, agarose gel extracted (NucleoSpin Extract II Kit, Macherey-Nagel GmbH & Co. KG, Düren, Germany) RT-PCR products ranging from 10^2^ to 10^8^ copies per sample were used as templates to generate standard curves. The plates contain duplicates of serially diluted samples for the standard curves and a no template control in duplicate. The reaction was quantified using SYBR-Green I in a reaction volume of 25 μl. RT-qPCR with Sybr Green I (1:40000) was performed under the following conditions: 95°C for 10min; 95°C for 30sec, 60°C (cortactin) or 64°C (GAPDH) for 1min and 72°C for 30sec, repeated 40 times and 72°C for 1min. Amplification was performed using 0.05 U/μl SureStart Taq DNA Polymerase in 1x Core PCR buffer with 2.5 mM MgCl_2_, 8.0% glycerol, 3% dimethyl sulphoxide (DMSO), 150nM of each primer [Cortactin: Forward: 5’- TTTCAAGAACACCAGACCCTCAA-3’; Reverse: 5’- CAAACTTCCCGCCATAACCATG-3’; 79 bp; GenBank accession number: XM_00563137; GAPDH: Forward: 5‘-GTCATCAACGGGAAGTCCATCTC-3‘; Reverse: 5‘-AACATACTCAGCACCAGCATCAC-3‘; 84 bp; GenBank accession number: AB038240], 30nM Rox as reference dye and 200μM dNTP mix.

Relative gene expression was normalised against the housekeeping gene GAPDH.

### Analysis of differentially expressed genes of published microarray data

For molecular characterisation of the potential influence of a persistent CDV infection on cellular motility, a data set obtained from a global gene expression analysis was used. Briefly, 4 replicates of non-infected and persistently CDV-Ond infected DH82 cells were analysed at 1d post seeding. RNA isolation was performed as described above and hybridized to Affymetrix Canine Genome 2.0 Arrays. Data sets are deposited in the ArrayExpress database (http://www.ebi.ac.uk/arrayexpress) under accession number E-MTAB-3942.

The present study focused on a list of manually selected genes associated with invadopodia (S1 Table) according to the literature [25,42–46]. Furthermore, a literature-based list of manually selected genes involved in EMT and MET, respectively, (S2 Table) was analogously analysed [32,71].

### Statistical analysis

Analysis of data not otherwise specified was performed using SAS Enterprise Guide (SAS-version 9.3; SAS Institute Inc, Cary, USA). The assumption of normality was tested using the Kolmogorov-Smirnov test and visual assessment of qq-plots of model residuals. In case of rejection of normal distribution, distribution-free nonparametric methods were applied. For descriptive statistics median and range were calculated. Used statistical procedures included the non-parametric Wilcoxon-Mann-Whitney two-sample-test for the analysis of the cell doubling assay, migration assay, immunofluorescence, TUNEL and RT-qPCR. Intracellular cortactin distribution was analysed using a three-way ANOVA. Proc mixed was used for the linear model. The level of significance was set at p ≤ 0.05 or p ≤ 0.01 for data with low ranges, respectively.

For microarray data, independent pair-wise Wilcoxon-Mann-Whitney tests (IBM SPSS Statistics version 20; IBM Corporation, Armonk, USA) were applied in order to compare the gene expression of non-infected and persistently CDV-Ond infected DH82 cells [72]. Significantly differentially expressed invadopodia-associated and EMT/MET-associated genes between persistently CDV-Ond infected and non-infected DH82 cells were selected employing a p-value ≤ 0.05 cut-off combined with a ≥ 2.0 or ≤ -2.0 fold change filter.

## Supporting Information

S1 Table 77 manually selected literature based genes, known to be involved in invadopodia formation and function. (DOC)

S2 Table 32 manually selected literature based genes, known to be involved in epithelial-mesenchymal transition (EMT) and mesenchymal-epithelial transition (MET). (DOCX)
