# Supplementary material for: Persistent Morbillivirus Infection Leads to Altered Cortactin Distribution in Histiocytic Sarcoma Cells with Decreased Cellular Migration Capacity
Source: PLoS One. 2016 Dec 2;11(12):e0167517. doi: 10.1371/journal.pone.0167517 (PMC5135102; doi:10.1371/journal.pone.0167517)
Supplement: S1 Table — (DOC) [file pone.0167517.s001.doc]

**Supplemental table S1: 77 manually selected literature based genes, known to be involved in invadopodia formation and function**

| Canine gene symbol | Gene Title (*Canis familiaris*) | Reference | p-value | Fold change |
| --- | --- | --- | --- | --- |
| A4GALT | alpha 1,4-galactosyltransferase | Linder 2009 | 0.057 | 1.318 |
| ABL1 | c-abl oncogene 1, non-receptor tyrosine kinase | Yamaguchi 2012; Murphy 2011 | 1.000 | 1.000 |
| ACTN1 | actinin, alpha 1 | Linder 2009; Murphy 2011 | 0.343 | 1.362 |
| ACTN2 | Actinin, alpha 2 | Linder 2009; Murphy 2011 | 0.029 | -1.731 |
| ACTN3 | actinin, alpha 3 | Linder 2009; Murphy 2011 | 0.686 | -1.067 |
| ACTN4 | actinin, alpha 4 | Linder 2009; Murphy 2011 | 0.029 | -1.399 |
| AFAP1 | actin filament associated protein 1 | Linder 2009; Murphy 2011 | 0.200 | -2.081 |
| ASAP1 | ArfGAP with SH3 domain, ankyrin repeat and PH domain 1 | Weaver 2006; Stylli 2008; Murphy 2011 | 0.029 | -1.406 |
| BCAR1 | breast cancer anti-estrogen resistance 1 | Murphy 2011 | 0.029 | -1.574 |
| CAV1 | caveolin 1, caveolae protein, 22kDa | Yamaguchi 2012 | 0.029 | -3.382 |
| CORO1C | coronin, actin binding protein, 1C | Murphy 2011 | 0.200 | -1.027 |
| CTTN | cortactin | Linder 2009; Weaver 2006; Stylli 2008; Revaeh 2014; Yamaguchi 2012; Murphy 2011 | 0.029 | -6.522 |
| Continuation supplemental table S1: | | | | |
| DNM1 | dynamin 1 | Weaver 2006; Stylli 2008; Revaeh 2014; Murphy 2011 | 0.686 | -1.033 |
| DNM2 | dynamin 2 | Weaver 2006; Stylli 2008; Revaeh 2014; Murphy 2011 | 0.057 | 1.246 |
| DNM3 | dynamin 3 | Weaver 2006; Stylli 2008; Revaeh 2014; Murphy 2011 | 1.000 | 1.000 |
| EGF | epidermal growth factor | Revaeh 2014; Murphy 2011 | 1.000 | 1.000 |
| EGFR | epidermal growth factor receptor | Weaver 2006; Revaeh 2014 | 0.057 | 1.277 |
| FAP | fibroblast activation protein, alpha | Linder 2009; Weaver 2006; Murphy 2011 | 1.000 | 1.002 |
| FGD1 | FYVE, RhoGEF and PH domain containing 1 | Yamaguchi 2012 | 0.057 | 1.328 |
| FSCN1 | fascin homolog 1, actin-bundling protein | Yamaguchi 2012; Murphy 2011 | 0.029 | 2.483 |
| FSCN2 | fascin homolog 2, actin-bundling protein, retinal | Yamaguchi 2012; Murphy 2011 | 1.000 | 1.000 |
| FSCN3 | fascin homolog 3, actin-bundling protein, testicular | Yamaguchi 2012; Murphy 2011 | 1.000 | 1.000 |
| GRB2 | growth factor receptor-bound protein 2 | Murphy 2011 | 0.029 | 1.700 |
| GSN | gelsolin | Linder 2009; Weaver 2006 | 0.029 | -1.820 |
| Continuation supplemental table S1: | | | | |
| IQGAP1 | IQ motif containing GTPase activating protein 1 | Revaeh 2014 | 0.057 | 1.371 |
| IQGAP2 | IQ motif containing GTPase activating protein 2 | Revaeh 2014 | 0.029 | -3.134 |
| IQGAP3 | IQ motif containing GTPase activating protein 3 | Revaeh 2014 | 1.000 | 1.000 |
| LIMK1 | LIM domain kinase 1 | Murphy 2011 | 1.000 | 1.000 |
| LIMK2 | LIM domain kinase 2 | Murphy 2012 | 0.343 | 1.155 |
| MAPK1 | mitogen-activated protein kinase 1 | Murphy 2011 | 0.686 | -1.090 |
| MET | met proto-oncogene (hepatocyte growth factor receptor) | Yamaguchi 2012 | 0.029 | -1.824 |
| MMP14 | matrix metallopeptidase 14 (membrane-inserted) | Weaver 2006; Revaeh 2014; Yamaguchi 2012; Murphy 2011 | 0.029 | 4.558 |
| MMP2 | matrix metallopeptidase 2 (gelatinase A, 72kDa gelatinase, 72kDa type IV collagenase) | Linder 2009; Weaver 2006; Revaeh 2014; Murphy 2011 | 1.000 | 1.028 |
| MMP9 | matrix metallopeptidase 9 (gelatinase B, 92kDa gelatinase, 92kDa type IV collagenase) | Linder 2009; Weaver 2006; Revaeh 2014; Murphy 2011 | 0.029 | 1.549 |
| MYH10 | myosin, heavy chain 10, non-muscle | Linder 2009; Murphy 2011 | 1.000 | 1.000 |
| MYH9 | myosin, heavy chain 9, non-muscle | Linder 2009; Murphy 2011 | 0.343 | -1.317 |
| Continuation supplemental table S1: | | | | |
| NCK2 | NCK adaptor protein 2 | Murphy 2011 | 1.000 | -1.000 |
| PDGFA | platelet-derived growth factor alpha polypeptide | Murphy 2011 | 0.029 | 2.089 |
| PDGFB | platelet-derived growth factor beta polypeptide | Murphy 2011 | 0.686 | 1.009 |
| PDGFC | platelet derived growth factor C | Murphy 2011 | 0.029 | 6.578 |
| PDGFD | platelet derived growth factor D | Murphy 2011 | 1.000 | 1.000 |
| PDGFRA | platelet-derived growth factor receptor, alpha polypeptide | Yamaguchi 2012 | 0.686 | -1.018 |
| PDGFRB | platelet-derived growth factor receptor, beta polypeptide | Yamaguchi 2013 | 1.000 | 1.000 |
| PIK3C2A | phosphoinositide-3-kinase, catalytic, alpha polypeptide | Yamaguchi 2012 | 0.029 | 1.348 |
| PIK3C2B | phosphoinositide-3-kinase, class 2, alpha polypeptide | Linder 2009; Murphy 2011 | 1.000 | 1.000 |
| PIK3C2G | phosphoinositide-3-kinase, class 2, beta polypeptide | Linder 2009; Murphy 2011 | 1.000 | 1.000 |
| PIK3C3 | phosphoinositide-3-kinase, class 2, gamma polypeptide | Linder 2009; Murphy 2011 | 0.057 | -1.137 |
| PIK3CA | phosphoinositide-3-kinase, class 3 | Linder 2009; Murphy 2011 | 0.200 | 1.119 |
| Continuation supplemental table S1: | | | | |
| PIK3CB | phosphoinositide-3-kinase, catalytic, beta polypeptide | Linder 2009; Murphy 2011 | 0.114 | 1.253 |
| PIK3CD | phosphoinositide-3-kinase, catalytic, delta polypeptide | Linder 2009; Murphy 2011 | 0.029 | 2.134 |
| PIK3CG | phosphoinositide-3-kinase, catalytic, gamma polypeptide | Linder 2009; Murphy 2011 | 0.029 | 3.168 |
| PIK3R1 | phosphoinositide-3-kinase, regulatory subunit 1 (alpha) | Linder 2009; Murphy 2011 | 0.114 | 1.228 |
| PLAUR | plasminogen activator, urokinase receptor | Murphy 2011 | 0.029 | 2.024 |
| PTK2 | PTK2 protein tyrosine kinase 2 | Linder 2009; Weaver 2006; Danielle 2011; Stylli 2008; Murphy 2011 | 0.029 | -3.226 |
| PTK2B | PTK2B protein tyrosine kinase 2 beta | Murphy 2011 | 0.686 | -1.051 |
| PXN | paxillin | Linder 2009; Stylli 2008; Revaeh 2014; Murphy 2011 | 1.000 | 1.009 |
| RAB8A | RAB8A, member RAS oncogene family | Revaeh 2014 | 0.486 | 1.052 |
| ROCK1 | Rho-associated, coiled-coil containing protein kinase 1 | Linder 2009 | 0.029 | 1.171 |
| ROCK2 | Rho-associated, coiled-coil containing protein kinase 2 | Linder 2009 | 0.114 | 1.712 |
| Continuation supplemental table S1: | | | | |
| ROS1 | c-ros oncogene 1 , receptor tyrosine kinase | Murphy 2011 | 1.000 | 1.000 |
| S100A4 | S100 calcium binding protein A4 | Murphy 2011 | 0.200 | -1.115 |
| SH3GL1 | SH3-domain GRB2-like 1 | Stylli 2008 | 0.886 | 1.114 |
| SH3GL2 | SH3-domain GRB2-like 2 | Stylli 2008 | 0.686 | -1.083 |
| SH3KBP1 | SH3-domain kinase binding protein 1 | Stylli 2008 | 0.029 | 1.915 |
| SH3PXD2A | SH3 and PX domains 2A | Linder 2009; Stylli 2008; Revaeh 2014; Yamaguchi 2012; Murphy 2011 | 1.000 | 1.025 |
| SH3PXD2B | SH3 and PX domains 2B | Murphy 2011 | 0.486 | -1.139 |
| SRC | v-src sarcoma (Schmidt-Ruppin A-2) viral oncogene homolog (avian) | Linder 2009; Weaver 2006; Stylli 2008; Revaeh 2014; Yamaguchi 2012; Murphy 2011 | 0.686 | -1.065 |
| SYNJ2 | synaptojanin 2 | Stylli 2008 | 1.000 | -1.005 |
| TGFB2 | transforming growth factor, beta 2 | Linder 2009; Murphy 2011 | 0.029 | -3.204 |
| TGFB3 | transforming growth factor, beta 3 | Linder 2009; Murphy 2011 | 0.686 | 1.066 |
| TRPM7 | transient receptor potential cation channel, subfamily M, member 7 | Murphy 2011 | 0.343 | 1.050 |
| Continuation supplemental table S1: | | | | |
| VAMP7 | vesicle-associated membrane protein 7 | Linder 2009 | 0.686 | -1.036 |
| VASP | vasodilator-stimulated phosphoprotein | Weaver 2006 | 0.029 | 1.602 |
| WAS | Wiskott-Aldrich syndrome (eczema-thrombocytopenia) | Linder 2009 | 1.000 | 1.000 |
| WASL | Wiskott-Aldrich syndrome-like | Weaver 2006; Revaeh 2014; Yamaguchi 2012; Murphy 2011 | 0.057 | -1.369 |
| WIPF1 | WAS/WASL interacting protein family, member 1 | Stylli 2008; Revaeh 2014; Murphy 2011 | 0.057 | 1.522 |
| ZYX | zyxin | Murphy 2011 | 1.000 | 1.000 |
