# Supplementary material for: Persistent Morbillivirus Infection Leads to Altered Cortactin Distribution in Histiocytic Sarcoma Cells with Decreased Cellular Migration Capacity
Source: PLoS One. 2016 Dec 2;11(12):e0167517. doi: 10.1371/journal.pone.0167517 (PMC5135102; doi:10.1371/journal.pone.0167517)
Supplement: S2 Table — (DOCX) [file pone.0167517.s002.docx]

| **Gene Symbol (Canis familiaris)** | **Gene Title (Canis familiaris)** | **Reference** | **p-value** | **Fold change** |
| --- | --- | --- | --- | --- |
| APC | adenomatous polyposis coli | Yang et al., 2014 | 0.686 | -1.060 |
| APC2 | adenomatosis polyposis coli 2 | Yang et al., 2014 | 1.000 | 1 |
| CD34 | CD34 molecule | Yang et al., 2014 | 0.029 | -1.099 |
| CD44 | CD44 molecule (Indian blood group) | Yang et al., 2014 | 0.029 | 2.657 |
| CDH1 | cadherin 1, type 1, E-cadherin (epithelial) | Yang et al., 2014; Somarelli et al., 2016 | 0.686 | 1.001 |
| CDH11 | cadherin 11, type 2, OB-cadherin (osteoblast) | Yang et al., 2014 | 1.000 | 1 |
| CDH12 | cadherin 12, type 2 (N-cadherin 2) | Yang et al., 2014 | 1.000 | 1 |
| CDH2 | cadherin 2, type 1, N-cadherin (neuronal) | Yang et al., 2014 | 0.029 | 77.297 |
| CLDN1 | claudin 1 | Yang et al., 2014 | 0.686 | 1.002 |
| CTNNB1 | catenin (cadherin-associated protein), beta 1, 88kDa | Yang et al., 2014 | 0.029 | -1.293 |
| DES | desmin | Yang et al., 2014 | 0.686 | 1.068 |
| EZH2 | enhancer of zeste homolog 2 (Drosophila) | Yang et al., 2014 | 0.029 | -1.597 |
| EZH2 | enhancer of zeste homolog 2 (Drosophila) | Yang et al., 2014 | 0.029 | -1.597 |
| GRHL2 | grainyhead-like 2 (Drosophila) | Somarelli et al., 2016 | 0.886 | -1.072 |
| HGF | hepatocyte growth factor (hepapoietin A; scatter factor) | Yang et al., 2014 | 0.029 | 2.766 |
| LEF1 | lymphoid enhancer-binding factor 1 | Yang et al., 2014 | 0.029 | -2.292 |
| NET1 | neuroepithelial cell transforming 1 | Yang et al., 2014 | 0.686 | -1.163 |
| NTN1 | netrin 1 | Yang et al., 2014 | 1.000 | 1 |
| OCLN | occludin | Yang et al., 2014 | 0.114 | 1.088 |
| SMAD3 | SMAD family member 3 | Yang et al., 2014 | 1.000 | 1 |
| SNAI1 | snail homolog 1 (Drosophila) | Yang et al., 2014; Somarelli et al., 2016 | 1.000 | 1 |
| SNAI2 | snail homolog 2 (Drosophila) | Yang et al., 2014; Somarelli et al., 2016 | 0.029 | 1.445 |
| SYP | synaptophysin | Yang et al., 2014 | 1.000 | 1 |
| TCF4 | transcription factor 4 | Yang et al., 2014 | 0.029 | 1.692 |
| TGFBR2 | transforming growth factor, beta receptor II (70/80kDa) | Yang et al., 2014 | 0.029 | -1.303 |
| TJP1 | tight junction protein 1 (zona occludens 1) | Yang et al., 2014 | 0.029 | -1.382 |
| TRPS1 | trichorhinophalangeal syndrome I | Yang et al., 2014 | 0.029 | 2.092 |
| TWIST1 | twist homolog 1 (Drosophila) | Yang et al., 2014; Somarelli et al., 2016 | 0.029 | 3.035 |
| TWIST2 | twist homolog 2 (Drosophila) | Somarelli et al., 2016 | 1.000 | 1 |
| VIM | vimentin | Yang et al., 2014 | 0.029 | -1.033 |
| ZEB1 | zinc finger E-box binding homeobox 1 | Yang et al., 2014; Somarelli et al., 2016 | 0.114 | 1.345 |
| ZEB2 | zinc finger E-box binding homeobox 2 | Yang et al., 2014; Somarelli et al., 2016 | 0.029 | 1.476 |
